# Vehicle Make and Model Recognition Using Bag of Expressions

**DOI:** 10.3390/s20041033

**Published:** 2020-02-14

**Authors:** Adeel Ahmad Jamil, Fawad Hussain, Muhammad Haroon Yousaf, Ammar Mohsin Butt, Sergio A. Velastin

**Affiliations:** 1 Centre for Computer Vision Research, Department of Computer Engineering, University of Engineering and Technology, Taxila 47050, Pakistanfawad.hussain@uettaxila.edu.pk (F.H.); ammarmohsin10@live.com (A.M.B.); 2 Swarm Robotics Lab, National Centre for Robotics and Automation, University of Engineering and Technology, Taxila 47050, Pakistan; 3 School of Electronic Engineering and Computer Science, Queen Mary University of London, London E1 4NS, UK; 4 Zebra Technologies Corp., London SE1 9LQ, UK; 5 Department of Computer Science and Engineering, University Carlos III de Madrid, 28270 Colmenarejo, Spain

**Keywords:** bag of expressions, intelligent transportation, make and model recognition, multiclass linear support vector machines, vehicular surveillance

## Abstract

Vehicle make and model recognition (VMMR) is a key task for automated vehicular surveillance (AVS) and various intelligent transport system (ITS) applications. In this paper, we propose and study the suitability of the bag of expressions (BoE) approach for VMMR-based applications. The method includes neighborhood information in addition to visual words. BoE improves the existing power of a bag of words (BOW) approach, including occlusion handling, scale invariance and view independence. The proposed approach extracts features using a combination of different keypoint detectors and a Histogram of Oriented Gradients (HOG) descriptor. An optimized dictionary of expressions is formed using visual words acquired through k-means clustering. The histogram of expressions is created by computing the occurrences of each expression in the image. For classification, multiclass linear support vector machines (SVM) are trained over the BoE-based features representation. The approach has been evaluated by applying cross-validation tests on the publicly available National Taiwan Ocean University-Make and Model Recognition (NTOU-MMR) dataset, and experimental results show that it outperforms recent approaches for VMMR. With multiclass linear SVM classification, promising average accuracy and processing speed are obtained using a combination of keypoint detectors with HOG-based BoE description, making it applicable to real-time VMMR systems.

## 1. Introduction

Vehicle make and model recognition has emerged as a prominent problem in the computer vision domain. There are many applications of VMMR in intelligent transport systems, including vehicle monitoring, intelligent parking systems, searching suspicious vehicles, electronic toll collection, autonomous vehicle systems, traffic analysis and so on. Another significant application of VMMR is vehicular surveillance in sensitive security premises; e.g., parking lots of airports, universities, stadiums and malls. An effective vehicle recognition approach is essential for acquiring high recognition accuracy. However, it is still a challenging task to recognize vehicle make and model in uncontrolled environments due to occlusions, scale variance, specific classification problems and view dependencies. In a traditional system, VMMR is based on a license plate recognition system or manual human observations [1]. These two techniques have a low success rate and many limitations. For a human observer, it is a laborious and time-consuming activity to remember and recognize a variety of vehicle makes and models efficiently. License plate recognition systems have proven to be robust to various lighting conditions with high processing speed and reasonable computational complexity [2]. On the other hand, a VMMR based on a license plate recognition system can produce incorrect results due to forgery, ambiguity, duplication or damaged license plates. Due to weaknesses in a traditional vehicle recognition system, computer-vision-based automated vehicle make and model classification have gained attention to improve an intelligent transportation system.

Vehicle make and model (VMM) identification methods fall into three main categories in the literature: feature-based, appearance-based and model-based methods. Feature-based methods identify vehicle models based on global or local invariant features. Hence, their performance depends on the reliability of features. For VMM, these have included symmetrical sped up robust features (SURF) [3], edge map [4], sparse representation [5] and probabilistic feature grouping [6]. Hsieh et al. [3] proposed a VMMR system using HOG and symmetrical SURF. Kumar et al. [7] detected vehicle logos by combining appearance-based and feature-based methods, and then using an SVM classifier to classify vehicles into four categories. Appearance-based methods classify vehicles by their inherent features containing shapes [8], symmetry [9], textures and dimensions [10]. Variation in the pose or change in the position of a camera can reduce the performances of those methods wherein the pose of vehicles is estimated using shape features, as in [9], but the authors do not discuss occlusion, lighting changes or viewpoint changes. Model-based recognition of vehicles includes the adaptive model [11], the approximate model [12] and the 3D model [11,13]. In [11], to classify vehicles, a 3D shape deformable vehicle model is used to detect different vehicle parts and then to recover shape information.

In this paper, we have opted for a feature-based method which is accurate and computationally fast. In this class of methods, VMMR systems typically include the following modules: (a) vehicle detection, (b) feature extraction and representation and (c) classification. Vehicle detection is a key step for VMMR and has been investigated by many researchers. Faro et al. [14] implemented a background subtraction procedure to subtract the vehicle region from the road, and then used a segmentation technique to eliminate full and partial occlusions among blobs of the vehicle. In [15], Jazayeri et al. used a hidden Markov model to probabilistically model the motion of the vehicle, and then to differentiate vehicles from the road. However, such kinds of motion features are not applicable for single motionless images. Because of color variation in vehicles, different color-based techniques have been used to detect vehicles [4,16,17]. Chen et al. [18], proposed a symmetrical points projection approach for the detection of vehicles from the background region.

Recognition of inherent vehicle properties for VMM is useful for offline or online vehicle identification, as compared to traditional license plate identification. To describe VMM on the basis of the regions of interest, numerous local features are extracted, with or without representing local features, into global features representations. The scale invariant feature transform (SIFT) [19] has been applied in different VMMR works, such as [20,21,22], but the SIFT descriptor is computationally slow. Baran et al. [20] used SIFT and SURF features and then embed them in a dictionary-based sparse vector of occurrence sums. In [21] Fraz et al. extracted SIFT features to create a lexicon that includes features of all the training images as words and then represents them by a fisher-encoded-based mid-level representation. Manzoor et al. [22] used HOG and SIFT for feature extraction. In [1], local features were extracted on the basis of Harris corners and gradient responses, and then locally normalized Harris strengths (LNHS) or square-mapped gradients (SMG) were used for global feature representation. He et al. [23], used edges and gradient-based features for feature extraction. For higher processing speed, the SURF descriptor [24] has gained the attention of many researchers and has been used in different works, such as [3,25]. Heish et al. [3] proposed SURF to extract vehicle features in a grid-wise manner and to represent them into grid-based global representation. In [25], Siddiqui et al. also used SURF for feature extraction, and then adopted a BOW-based approach for global representation. Two different techniques have been proposed for dictionary building in [25]: modular dictionary and single dictionary. In [26,27] Nazir et al. proposed the dynamic spatio-temporal bag of expressions (D-STBoE) model and the BoE framework for action recognition which improves the existing strength of bag of words. A global feature ensemble representation is discussed by Chen et al. [18] who combined the HOG vehicle features extracted in a grid-based pattern.

In computer vision, numerous classification approaches have been used to improve the VMMR classification process significantly. Psyllos et al. [28], apply a probabilistic neural network for classification on the SIFT features extracted from vehicle images. A naive Bayes classifier is used for the classification of VMM in [1]. For classification purposes, [23] reported the use of an ensemble of neural networks as well as AdaBoost, SVM, and KNN. Varjas et al. [29] also proposed a KNN classifier with an enhancement of a correlation-based distance metric. A euclidean distance-based matching scheme is reported in [21], but it is a time-consuming task to match in a brute force manner. For the classification of VMM, hamming distance and sparse representation approaches have been used in [18]. In [22], random forest classification is used for the recognition of VMM while Tang et al. [30] proposed a nearest neighborhood classification approach for the recognition of vehicles. Jie Fang et al. [31] and Afshin Dehghan et al. [32] describe a convolutional neural network (CNN) for the classification of a vehicle’s make and model. Random forest and SVM are suggested for recognition of VMM in [33]. Indeed, SVM-based classification schemes have been applied in different VMMR systems such as [3,13,20,25]. In [34], the authors use a PCANet-based CNN for the recognition of a vehicle make-model.

**Figure 1:**
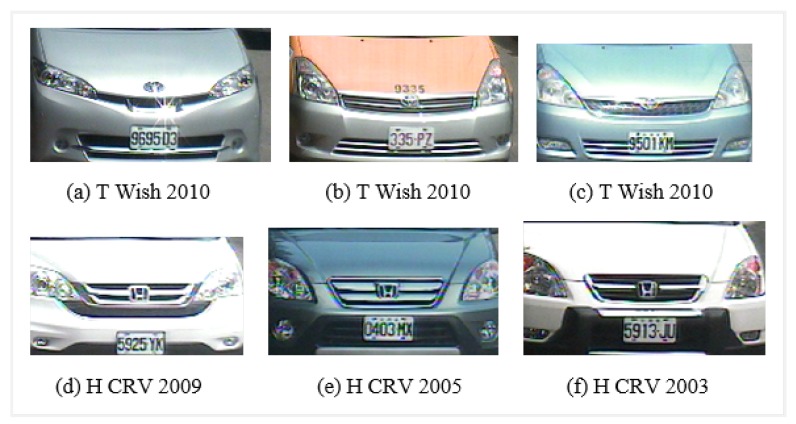
Multiplicity problems with (**a**–**c**) Toyota Wish (C4) and (**d**–**f**) Honda CRV (C11) in the NTOU-MMR dataset. The multiplicity problem means one VMM often displays different shapes due to different manufacturing years.

**Figure 2:**
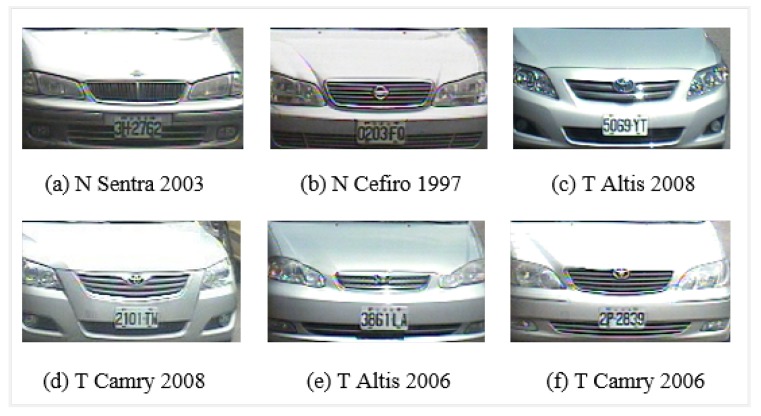
Intra-make ambiguity problems between (**a**) Nissan Sentra 2003 and (**b**) Nissan Cefiro 1997; (**c**) Toyota Altis 2008 and (**d**) Toyota Camry 2008; and (**e**) Toyota Altis 2006 and (**f**) Toyota Camry 2006 in the NTOU-MMR dataset. Intra-make ambiguity results when different vehicles (models) from the same company (make) have a comparable shape or appearance.

**Figure 3:**
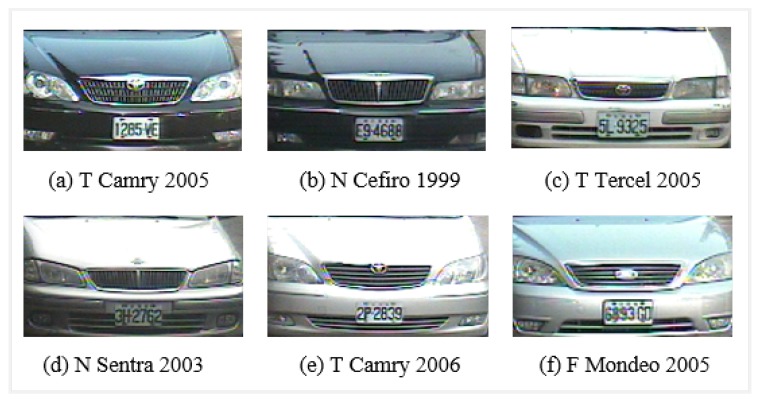
Inter-make ambiguity problems between (**a**) Toyota Camry 2005 and (**b**) Nissan Cefiro 1999; (**c**) Toyota Tercel 2005 and (**d**) Nissan Sentra 2003; and (**e**) Toyota Camry 2006 and (**f**) Ford Mondeo 2005 in the NTOU-MMR dataset. The inter-make ambiguity problem refers to the case where different vehicles (models) manufactured by different companies (makes) can have comparable appearances or shapes.

Some of the problems related to VMMR tasks are: varying image quality, variations in lighting conditions, changes in weather conditions (rainy day, sunny day and night), viewpoint changes, perspective distortion, etc. Shadows, backlighting conditions and occlusions can significantly change visual properties. There are considerable variations in the size, color, orientation, pose and shape between vehicles. Another major challenge in VMMR is multiclass classification. In this scenario, there are two main categories: (a) multiplicity and (b) ambiguity. The former refers to the issue of vehicle models of one make (company) having different shapes as illustrated in Figure 1; e.g., the “Wish” models manufactured in 2010, 2009 and 2005 by “Toyota” have different visual appearances due to shape upgrades. Similarly, the “CRV” models of the “Honda” make pose the same challenge. The problem of ambiguity is further divided into two sub-problems: (a) intra-make ambiguity and (b) inter-make ambiguity. In the first case, a problem occurs when different models of the same make show similar visual appearances, as illustrated in Figure 2; i.e., the “Sentra” and “Cefiro” models of the “Toyota” make have comparable front faces which highlights the intra-make ambiguity. In the second case, problems arise when vehicle models of different makes have visually comparable rear/front views, as illustrated in Figure 3; e.g., the “Toyota Camry 2005” and the “Nissan Cefiro 1999” make and model have similar front faces, and the same trend is seen in other makes and models.

To overcome the above-mentioned problems in VMMR, this paper presents:

- An evaluation of different combinations of feature keypoint detectors and the HOG descriptor for feature extraction from vehicle images.
- A global dictionary building scheme to tackle the ambiguity and multiplicity problems for vehicle make and model recognition. The optimal size of the dictionary is investigated by a series of experiments.
- An evaluation of a previously unexplored approach, “bag of expressions,” for VMMR. On the basis of BoE, a multiclass linear SVM classifier was trained for classification. Contributions to VMMR work include learning visual words from a specific class with BoE features enhancement and an improvement in performance.

The superiority and effectiveness of the bag of expression approach for vehicle make and model recognition over the state-of-the-art methods are tested on random training-testing dataset splittings of the NTOU-MMR dataset [18].

## 2. Proposed Methodology

This paper proposes and evaluates an up till now unexplored approach for VMMR based on BoE and multiclass linear SVM. The key phases of the approach are as follows: (1) feature point extraction (using the KAZE (according to its originators a Japanese word that means wind [35]), SURF (Speeded Up Robust Features), FAST (Features from Accelerated Segments Test) and BRISK (Binary Robust Invariant Scalable Keypoints) feature detectors), (2) HOG-based description, (3) BoE-based feature representation and (4) linear SVM-based classification. The main focus of this work is on the frontal view of the vehicle for make and model recognition, because other parts, such as a hood or windshield, could mislead the classifier due to fewer variations across different vehicle makes and models. This is a typical approach in the literature.

**Figure 4:**
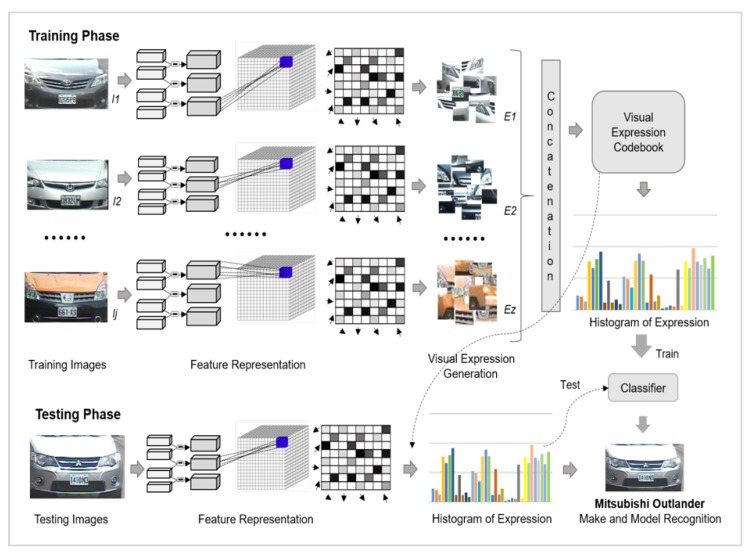
Bag of expressions-based framework for vehicle make and model recognition (VMMR).

The summary of the proposed VMMR approach is presented in Figure 4. In the training phase, features are first extracted from labeled images with specific vehicle class labels as $L=\{l_{1},l_{2},l_{3}...l_{j}\}$, where *j* is the total number of vehicle classes. During the next phase, a visual expression codebook is generated by defining visual words. A histogram of expressions is generated by counting the occurrences of every expression for the training images of a vehicle. Finally, a BoE-based representation of training images is applied for the training of a supervised classifier. During the testing phase, a feature representation is acquired for unlabeled vehicle images and quantized using a codebook of a visual expression generated in the training phase. Then, occurrences of each expression are added to build a histogram of expressions which is then passed to the trained classifier to detect the vehicle label. A more detailed explanation of these processes is given in the following subsections.

### 2.1. Feature Points Detection

**Figure 5:**
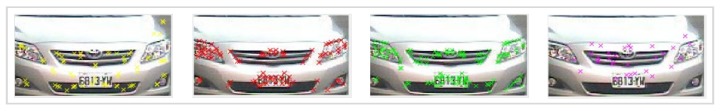
Detected feature points using KAZE, FAST, BRISK and SURF feature detectors respectively.

Different types of feature point detectors are applied to detect the unique feature points from each class of vehicle, as shown in Figure 5. In this work, the KAZE feature detector [35] has been used to detect feature points of interest from vehicle images. To detect the points of interest, the results of the scale-normalized determinant of the Hessian were calculated at different numbers of scale levels.

(1) $$L_{Hessian}=\sigma^{2}\left(L_{x}xL_{y}y-L_{x}^{2}y\right)$$

where $L_{x}x$ are the second order horizontal derivatives, $L_{y}y$ are the second order vertical derivatives and $L_{x}x$ is the second order cross derivative. From the nonlinear scale space $L^{i}$ the set of filtered images is given, the response of the detector was analyzed at different scale levels $\sigma_{i}$.

The FAST [36] detector has also been used in this work for feature points detection. FAST was the first AST (accelerated segment test)-based algorithm for feature detection and it first examines the values of the intensity function of pixels in a circle (of sixteen pixels) of radius *r* around the candidate point *p* to decide if a candidate point *p* is either a corner point or not. After comparing the intensity values of four pixels ($I_{1}$, $I_{5}$, $I_{9}$, $I_{1}3$) with the intensity of the candidate point *p*, if three of these four pixels meet the threshold criteria, then the candidate point *p* is selected as an interest point (corner). Conversely, if three of these four-pixel values are not above or below the intensity of candidate point *p* plus a threshold, then the candidate point *p* will not be a corner point. If they are above or below, then all sixteen pixels are examined. In this scenario at least twelve pixels should fall within the given criterion. The process is repeated for all remaining pixels of the image.

The BRISK [37] feature detector has also been studied here. BRISK is a binary descriptor, based on a FAST detector that makes computations directly on image patches. It includes three parts: (a) sampling pattern, (b) sampling pairs and (c) orientation compensation. BRISK uses a sampling pattern surrounding keypoints on a set of concentric circles to identify whether the points are corners in FAST or not. Then pairs are divided into two subsets, long distance and short distance pairs. For rotation invariance, the sum of a locally computed gradient is used between short distance and long distance pairs.

Finally, the SURF [24] detector has also been tested. The SURF detector is capable of producing interest points which are scale and rotation invariant. For keypoint detection, it uses integral images and a 2D Haar wavelet. SURF uses the sum of the 2D Haar wavelet response to find the keypoint detection around the region of interest. A Hessian matrix approximation is used to estimate the operators of Gaussian derivatives. The Haar wavelet approximation can be computed effectively on integral images without considering scale factor. For accurate localization of multiscale SURF features, interpolation is required. Because of the blob-like structure, its performance is dependent on non-maximal-suppression of the determinants of the Hessian matrices. A multiscale SURF features interpolation is essential for accurate localization.

### 2.2. Description of Features

The HOG descriptor was proposed by Dalal and Triggs [38]. It is applied after the extraction of keypoints, as described above, from the vehicle images. The descriptor was initially used for the detection of pedestrians from static images and it works by finding the frequency of gradient directions from the localized region in the input image. This image is divided into grid-like patterns to get local regions. It can represent the appearances and shapes of different objects from images with the help of gradient direction distributions. These distributions are computed by dividing the input image into grid-like patterns and then finding the gradient directions and gradient magnitudes from each grid pattern of the image. The histogram of gradients is formed by computing the gradient directions from each cell of grid in range of 0 to 180 degrees. The number of bins in the histogram is usually nine, each corresponding to 20 degrees intervals. For each pixel in the given cell, the magnitude of its gradient is added to a histogram bin as per the gradient direction. The histograms from these individual cells are then combined into 2 × 2 cells and concatenated into bigger blocks. The overall size of the HOG feature descriptor becomes 36 with four cells, each having a dimension of nine. The descriptor size is dependent on the resolution of the image, block size and cell size. Furthermore, to account for variations in brightness and contrast, each block of a histogram is normalized.

### 2.3. Generation of Visual Expressions

**Figure 6:**
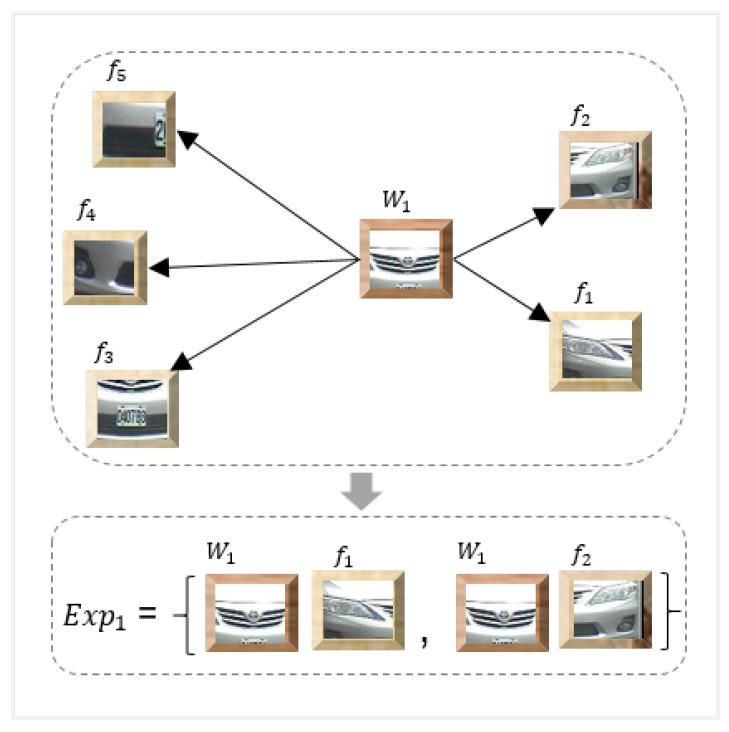
Visual expression generation (for illustrative purposes N has been set to 2 and only one visual word is considered).

For a given set of vehicle classes, the aim is to learn a global representation for the vehicle classes by means of singular dictionary generation. The obtained features are characterized as follows: $F=\{f_{1},f_{2},f_{3},\ldots \ldots ,f_{j}\}$ where *j* represents the number of vehicle classes. The clusters are formed by applying the widely used k-means clustering algorithm [39] on the obtained features, and distinctive visual words are obtained for all the vehicle classes. The centroids obtained by k-means represent the visual words which are denoted by *W*. The visual vocabulary is represented as $W=\{W_{1},W_{2},W_{3},\ldots \ldots ,W_{n}\}$ where *W* is a complete set of visual words for all the vehicle classes and n represents a total number of visual words. Each visual word from *W* is paired with its *N* nearest neighbors and forms an expression representation $Exp_{i}$, as shown in Figure 6.

For each visual word $W_{i}$ in a visual vocabulary *W*, we intend to find neighboring features that are closest to the visual word. These neighboring features are computed using a distance measure such as euclidean or Mahalanobis. In Figure 6, let us assume that the arrows denote distance, $W_{1}$ represents the visual word and that $f_{1},f_{2},f_{3},\ldots \ldots ,f_{5}$ represent the features considering N = 2; i.e., two nearest neighbors. The expression ${\left[Exp\right]}_{i}$ is formed by finding the closest features to the visual word. In this case, $W_{1}$ is nearest to two features ${\left[f\right]}_{1}$ and ${\left[f\right]}_{2}$. Therefore, $W_{1}$ is combined with ${\left[f\right]}_{1}$ and ${\left[f\right]}_{2}$ by finding the mean of each pair $\left(W_{1},{\left[f\right]}_{1}\right)$ and $\left(W_{1},{\left[f\right]}_{2}\right)$. These pairs are stacked together and added into a visual expression codebook. The process is repeated for the remaining visual words; thus, the final expression codebook contains the mean pairs $\left(W_{i},{\left[f\right]}_{i}\right)$ for each visual word.

For each of these visual words *w*, the neighboring features are computed through a distance measure to form an expression dictionary. In experimentation, different distance measures have been used for distance calculation, such as Mahalanobis and euclidean distances. The Mahalanobis distance is calculated as follows:

(2) $$D_{\omega \mu }=\sqrt{\left(\omega -\mu \right)S^{-1}{\left(\omega -\mu \right)}^{t}}$$

where $S^{-1}$ represents the inverse covariance matrix, ω represents a visual word and μ represents the feature distribution. The euclidean distance is given by the well-known expression

(3) $$D_{\omega \mu }=\sqrt{\left(\omega -\mu \right){\left(\omega -\mu \right)}^{t}}$$

Neighboring features are described in terms of independent pairs. These pairs are combined with the visual words irrespective of their relationship with other visual words. For example, a visual word having 10 neighboring features will be paired with each one to form 10 expressions. This offers some degree of view independent representation which is tolerant of occlusion. Thus, the achieved representation in terms of expressions provides discriminative power to word pairs to discard information relevant to other visual words and recognize the vehicle classes even in an occluded environment. The problem of view independence is addressed by forming a vector of frequency counts of expressions for each vehicle class. These vectors help in differentiating different makes and models. The expressions formed through visual word pairs are represented as follows:

(4) $$\begin{array}{cc} Exp_{1} & =\{exp_{1}1,exp_{1}2,exp_{1}3,\ldots \ldots ,exp_{1}N\}\\ Exp_{2} & =\{exp_{2}1,exp_{2}2,exp_{2}3,\ldots \ldots ,exp_{2}N\}\\ Exp_{3} & =\{exp_{3}1,exp_{3}2,exp_{3}3,\ldots \ldots ,exp_{3}N\}\\ \ldots & \\ Exp_{z} & =\{exp_{z}1,exp_{z}2,exp_{z}3,\ldots \ldots ,exp_{z}N\} \end{array}$$

where *N* represents the number of nearest neighboring features combined to form an expression. Furthermore, these expressions are combined together to form an expression dictionary $E=\{Exp_{1},Exp_{2},Exp_{3},\ldots \ldots ,Exp_{z}\}$ where *z* is the total number of expressions. The nearest features combined with visual words are expected to vary distinctively with respect to each class; hence, the formed expressions are discriminative enough to distinguish between different classes.

### 2.4. Histogram of Expressions

**Figure 7:**
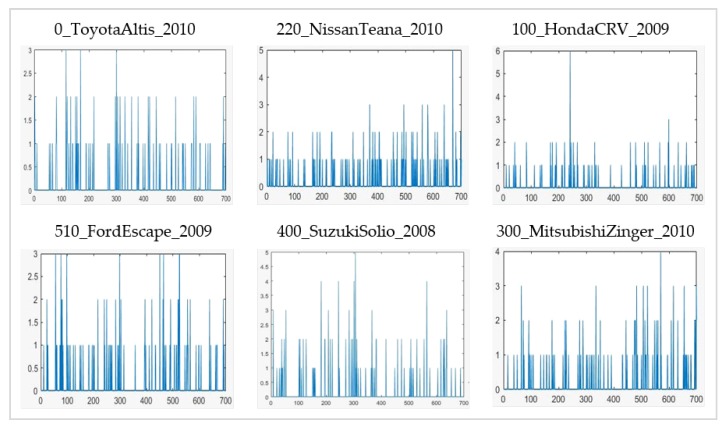
Histograms of expressions for the NTOU-MMR dataset (random sample of each specified vehicle class).

Histograms of expressions are generated by computing the frequency of occurrence of each given expression from each of the feature vectors of the image. This mapping of features to neighboring expressions is computed through a distance measure; e.g., euclidean distance or Mahalanobis distance. The histogram of expressions for vehicle images acts as the training or testing sample to be used in classification. The histograms of expressions displayed in Figure 7 belong to some of the vehicle samples taken from different classes of NTOU-MMR dataset. These are used as training and testing data for a classifier. The frequency of expressions varies for each class of the dataset which helps in differentiating different vehicle classes.

### 2.5. Classification

The identification of a vehicle manufacturer and model is an essential task when designing a VMMR system. The well-known multiclass linear SVM classifier for the classification of vehicle make and model has been applied. This procedure learns β and β(β−1)/2 binary SVMs, where β represents the total number of unique vehicle classes by applying one-versus-all and all-pairs coding design models respectively. By applying a sparse-random coding design model, this procedure learns random (but approximately 15 $log_{2}\beta$) binary SVMs. Different SVM parameters are chosen to achieve good recognition accuracy, as further discussed in the experimental discussion and results section below.

## 3. Experimental Results and Discussion

**Figure 8:**
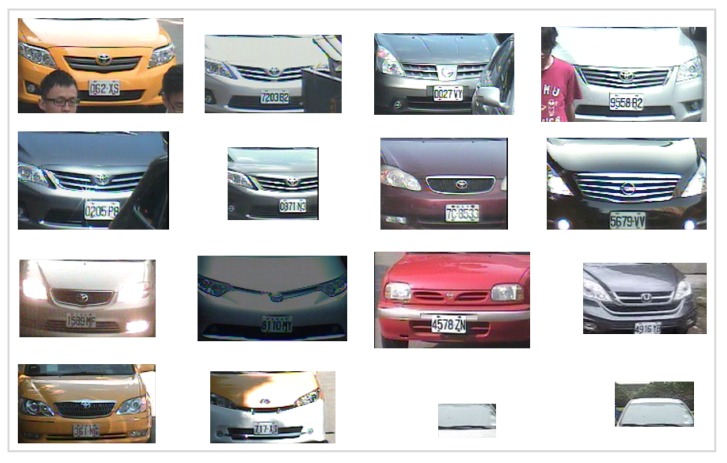
Some of the challenging scenarios in the NTOU-MMR dataset. The BoE-based approaches were successful in predicting the make–model class in all of the above cases except the last two (noisy images).

To investigate the efficiency and effectiveness of the BoE-based approaches for VMMR, the proposed approach has been evaluated on the publicly available NTOU-MMR dataset [18], as it serves as a good benchmark dataset for performance comparison among different VMMR works. This dataset was published in the relevant work of VMMR [3] and has been gathered under the Vision-based Intelligent Environment (VBIE) project [18]. The original dataset was captured in different environmental conditions and then the whole dataset was divided into a training image set and testing images set. The training images set contains 2846 vehicle images, and the testing set contains 3793 vehicle images. There are 29 different classes in the dataset. The set of make-model classes in NTOU-MMR dataset is: class number one (C1) Toyota Altis, C2 Toyota Camry, C3 Toyota Vios, C4 Toyota Wish, C5 Toyota Yaris, C6 Toyota Previa, C7 Toyota Innova, C8 Toyota SURF, C9 Toyota Tercel, C10 Toyota RAV, C11 Honda CRV, C12 Honda Civic, C13 Honda Fit, C14 Nissan March, C15 Nissan Livna, C16 Nissan Teana, C17 Nissan Sentra, C18 Nissan Cefiro, C19 Nissan Xtrail, C20 Nissan Tiida, C21 Mitsubishi Zinger, C22 Mitsubishi Outlander, C23 Mitsubishi Savrin, C24 Mitsubishi Lancer, C25 Suzuki Solio, C26 Ford Liata, C27 Ford Escape, C28 Ford Mondeo and C29 Ford Tierra. The dataset contains the images of the vehicles captured from different viewing angles in a range between −20${}^{\circ }$ to +20${}^{\circ }$ and in different weather conditions; for example, cloudy, rainy and sunny. Images of the dataset were also captured at night time, day time and under different illumination conditions. In addition, there are a few images of vehicles occluded by other objects, such as an umbrella, pedestrians and another vehicle. Some of the challenging scenarios in this dataset are shown in Figure 8.

There are some issues with the NTOU-MMR dataset: (1) duplication of images: in some classes, duplicated images of a vehicle saved with a different label; (2) misplacement of images: various class directories contain the vehicles of other classes; (3) biased division of testing and training images: the strategy for how to divide the data for each class into training and testing is not clear, but any division can affect the performance of the system; (4) noisy data: some vehicle image samples are noisy and also contain irrelevant data for processing. All the experiments reported here were performed on Intel Core™ i7-4600M CPU (2.90 GHz) 8 GB RAM, using MATLAB. Similar to prior VMMR work [25], for evaluation purposes we have used a hold-out cross validation scheme for the dataset [18] to build dissimilar training and testing splits for all vehicle classes. Ten such training and testing splits are built, and the mean accuracy of these splits is reported. For every split of the data set, randomly, 20% of vehicle images are picked for testing purposes and the other 80% of the vehicle images are used to train the algorithm—referred to as 80–20 NTOU-MMR splits. These proportions are commonly used by other researchers using this dataset. For each split, the division criteria for a number of images for training (#Train) and testing (#Test) sets are similar to [25]. Processing speed and mean accuracy are obtained by averaging results for every split. Results have also been evaluated with 70–30 and 60–40 splits.

### 3.1. Selection of Optimal Parameters

Using the 80–20 NTOU-MMR Datasets, optimized parameters for each phase were obtained by cross-validation. Here, the best results mean the best trade-off between average accuracy and processing speed.

#### 3.1.1. Dictionary Size

In the dictionary building phase, dictionary size (the number of clusters) affects the overall performance of the VMMR module in the context of processing speed and accuracy. For the KAZE HOG feature descriptor, dictionary size was varied from 100 to 1000 and the best results were achieved for dictionary size 350, as shown in Table 1. For the other combinations, dictionary size was varied in the range 100 to 3000, and it was found that the best trade-offs between accuracy and speed for dictionary size were 1200 for BRISK HOG, 2200 for SURF HOG and 1500 for FAST HOG, as presented in Table 2.

#### 3.1.2. BoE Parameters

**Figure 9:**
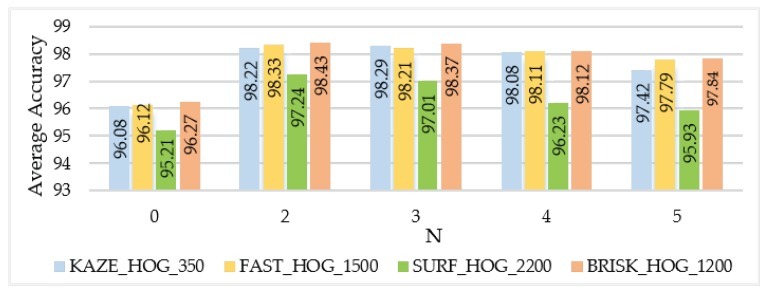
Average accuracy with respect to number of neighbors N for visual expression codebook creation for the NTOU-MMR dataset using the sparse-random coding model and Mahalanobis distance.

Optimization of BoE parameters involves some different parameters: Which distance measure is used for distance calculation and the number of nearest neighbors N for the formation of expression codebook. The number of nearest neighbors N was varied from 0 to 5 and the best results were obtained with N = 2, as shown in Figure 9. Euclidean and Mahalanobis distance measures were tested for codebook generation.

#### 3.1.3. SVM Parameters

Finally, optimized classifier parameters were obtained on the basis of optimal dictionary sizes. In the classification phase, the multiclass linear SVM classifier was applied with the following parameters: a linear kernel function; an automatic kernel scale; a size of box constraint set to 29; fit posterior and standardize enabled. Three different coding design models were used (one-vs.-all, sparse-random and all-pairs).

### 3.2. Performance Evaluation

Different experiments were carried out to study the effects of parameters to aim for an efficient VMMR framework. SURF-based BoE obtains 95.44% average accuracy with a processing speed 11.0 fps. KAZE-based BoE achieves 96.76% average accuracy and 16.6 fps. As explained earlier, different dictionary sizes were explored. Although the best accuracy is achieved with a larger dictionary size, computation time is sacrificed to the point where it is not suitable for real-time applications. Therefore, dictionary size was chosen to maintain reasonable speeds. For example, for KAZE HOG the approach was tested with dictionary sizes from 100 to 1000 (Table 1), obtaining 93.03% average accuracy with size 100 and 99.01% with size 1000. For the best trade-off between average accuracy and processing speed, a dictionary size of 350 was chosen, achieving average accuracy of 98.22%. For the FAST HOG, BRISK HOG and SURF HOG feature descriptors, dictionary size was varied from 100 to 3000 (Table 2); choosing a size of 1500, for example, for FAST HOG, achieves 98.33% average accuracy (even if with a size of 3000 and average accuracy of 98.86% is reached). For SURF HOG a best result of 97.24% was obtained with a dictionary size of 2200. For BRISK HOG, for the best trade-off between average accuracy and processing speed, a dictionary size of 1200 was chosen, achieving 98.43% average accuracy. Experiments with different validation splits were performed and are presented in Table 3. From these experiments, it can be concluded that the approach performs well also for the additional 70–30 and 60–40 splits.

Different combinations of distance measure and coding design model were evaluated with KAZE HOG, FAST HOG, BRISK HOG and SURF HOG, as presented in Table 4. In the case of KAZE HOG ( dictionary size = 350), the highest average accuracy (98.27%) was obtained with one-vs.-all and Mahalanobis, but processing speed was worse compared to the sparse-random and Mahalanobis combination. The same descriptor with all-pairs results in less accuracy as well as slower processing speed compared to other combinations. Its best result (best trade-off between speed and accuracy) is 98.22% average accuracy using sparse-random and Mahalanobis combination. FAST HOG (dictionary size = 1500), with one-vs.-all and euclidean results in the highest average accuracy (98.36%), but it is computationally slow compared to sparse-random. The best trade-off results (98.33%) are obtained using a combination of sparse-random and Mahalanobis with dictionary size 1500. For SURF HOG feature descriptor (dictionary size = 2200), its highest accuracy is 97.32%, using euclidean and sparse-random, but at the expense of computational speed. The best trade-off performance is achieved using a sparse-random and Mahalanobis distance combination, reaching 97.24%. BRISK HOG (dictionary size = 1200), reaches its highest average accuracy at 98.49% using one-vs.-all and Mahalanobis, but again sacrificing speed. Its best trade-off result is 98.43% using Mahalanobis and sparse-random combination. The average per-class accuracies of the proposed approaches on the NTOU-MMR dataset are presented in Table 5, which illustrates the class-wise dominance of the proposed approaches on NTOU-MMR dataset.

**Figure 10:**
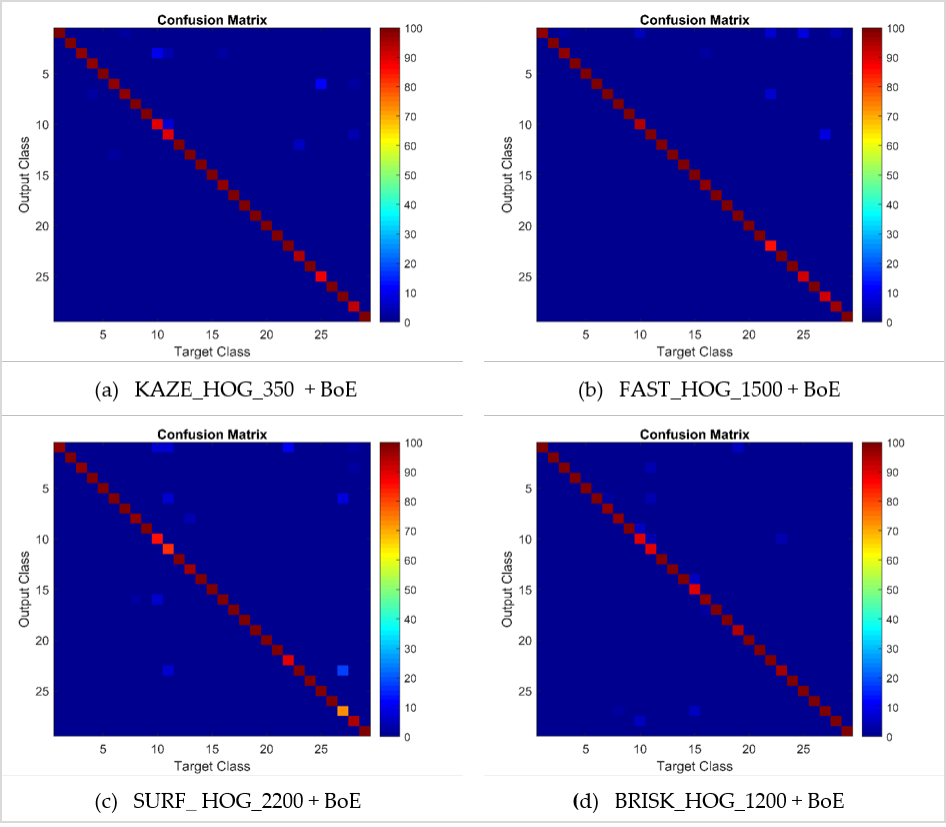
Confusion matrices for bag of expression for the 80–20 NTOU-MMR dataset.

**Figure 11:**
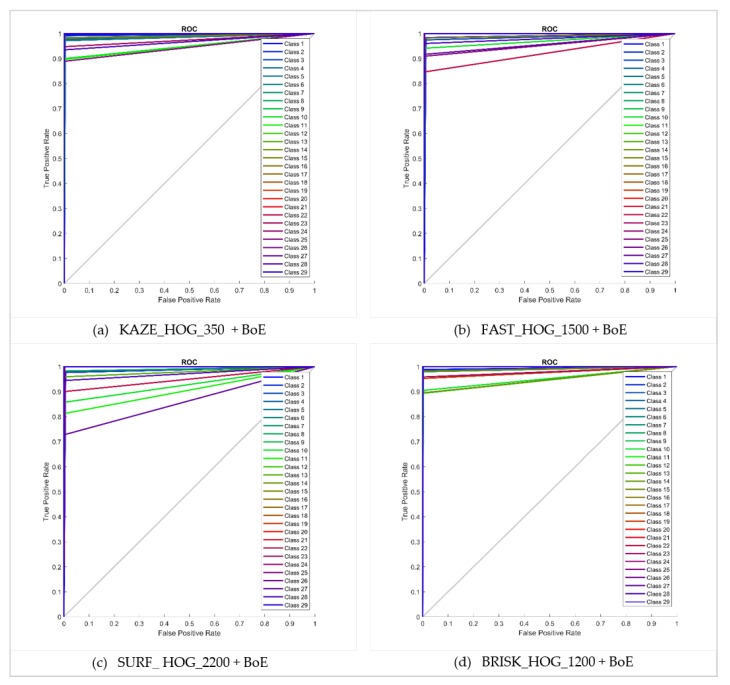
ROC curves for bag of expression for 80–20 NTOU-MMR dataset.

The performance of the approach presented here is also illustrated by means of confusion matrices and ROC curves, as shown in Figure 10 and Figure 11 respectively. The problem of multiplicity, as discussed in Figure 1, was resolved by applying the proposed approaches. For example, C4 had a multiplicity problem that did not degrade the results, as can be seen in Table 5. The multiplicity problem in C11 (Honda CRV) did not significantly affect the FAST HOG-based BOE approach. However, KAZE, SURF and BRISK degrade somewhat to 90.4%, 82.4% and 89.6% average accuracy. The inter-make ambiguity problems between C2 and C18; C9 and C17; and C2 and C28, as shown in Figure 3, were also addressed. The intra-make ambiguity problems between C17 and C18; C1 and C2; and C1 and C2, as shown in Figure 2, were also overcome. The results for all these ambiguous classes show good performance, as can be seen in Table 5. Furthermore, these good performance indicators show that the proposed approach is able to cope well with many of the challenges in this dataset (as illustrated by Figure 8).

### 3.3. Computational Cost

The proposed approach not only results in higher accuracy but also good time performance, to levels suitable for real-time operation compared to state-of-the-art work, even with relatively modest hardware (please see Table 6). For example, in our case, KAZE HOG with a dictionary size of 350, achieved an average accuracy of 98.22% with a processing speed 24.7 fps. FAST with a dictionary size of 1500, obtains a processing speed of 15.4 fps with an average accuracy of 98.33%. SURF with a dictionary of size 2200, has an average accuracy of 97.24% with 18.6 fps. Finally, BRISK with a dictionary size of 1200, achieves 6.7 fps with the highest average accuracy of 98.43%.

### 3.4. Comparison with State-Of-The-Art

The proposed BoE-based approaches for a vehicle make and model recognition presented in this paper outperform numerous related VMMR works, both in terms of classification accuracy and processing speed. Accuracy and speed comparisons are presented in Table 6. Hardware used in other works, where known, is also given. The table provides comprehensive results on NTOU-MMR and other VMMR datasets. It is pertinent to mention that other datasets are quite similar to NTOU-MMR in terms of environment and some of the challenges discussed earlier. Furthermore, all these methods mainly focus on frontal view of vehicles for VMMR. The performance and computational cost of [18] on the NTOU-MMR dataset are not sufficient for real-time VMMR. The processing speed of [20,23,30] is not up-to-the-mark for a real-time environment. The accuracy obtained from these works is also not high enough for a high performance VMMR system. The performance of [31,32] is sufficient for VMMR work but the authors do not discuss the computational cost in their work. The performance and computational cost of [40,41] are sufficient for VMMR work but less than what has been presented here. The work proposed in [42] does not provides satisfactory results on the NTOU-MMR dataset. The performance and computational cost of [25,33] on the NTOU-MMR dataset are acceptable but far behind from the proposed approach in terms of average accuracy and processing speed. Therefore, it can be concluded from Table 6 that the BoE-based VMMR procedure is superior in the context of performance and computational cost.

## 4. Conclusions

This paper has presented a new BoE-based approach for the recognition of vehicle make and model with a higher degree of accuracy which is also suitable for real-time ITS applications. This work has determined, from the quality measures, that the KAZE HOG-based BoE combination is more suitable for a real-time VMMR environment. An expressions-based global dictionary building scheme has been studied to address the multiplicity and ambiguity problems in VMMR. From experimental evaluations, optimal sizes for the dictionaries have been recommended. Optimal parameter selection and evaluation of SVM classification has proven useful for real-time VMMR.

The superiority and effectiveness of the BoE-based approach over the state-of-the-art methods have been validated using random training-testing splits of the NTOU-MMR Dataset. Although the proposed approaches achieved good performances, we plan to explore more robust features in the future that are applicable for real-time VMMR. We will also explore further an encoding scheme and a classification scheme. Likewise, the learning approach also needs to be enhanced when unseen vehicle models are included in the dataset. We will also explore different schemes of ROI (Region of Interest) utilization and division. We also have a plan to build a general VMMR dataset for VMMR work which satisfies the requirements of the standard benchmark dataset. Exploring deep learning features from a pretrained network instead of HOG with a bigger dataset is also in line for future work. 

## Figures and Tables

**Table 1:** Impact of varying dictionary size on average accuracy with the KAZE feature detector (using Mahalanobis and sparse-random combination).

Dictionary Size	100	200	300	350	400	500	600	700	1000
KAZE_HOG _ BoE	93.03	96.36	97.93	98.22	98.39	98.58	98.74	98.88	99.01

**Table 2:** Impact of varying dictionary size on average accuracy with FAST, BRISK and SURF feature detectors (using Mahalanobis and sparse-random combination).

Dictionary Size	FAST_HOG_BoE	SURF_HOG_BoE	BRISK_HOG_BoE
100	90.56%	86.54%	91.65%
500	96.67%	93.68%	97.59%
1000	97.56%	95.93%	98.21%
1200	97.94%	96.11%	98.43%
1500	98.33%	96.26%	98.76%
2000	98.65%	97.08%	98.94%
2200	98.73%	97.24%	99.06%
2500	98.78%	97.66%	99.09%
3000	98.86%	97.71%	99.14%

**Table 3:** Impact of validation splits on average accuracy for KAZE, FAST, BRISK and SURF (using Mahalanobis and sparse-random combination).

Feature Extraction		Average Accuracy % with Validation Scheme		
		Mahalanobis + sparse-random	Mahalanobis + sparse-random	Mahalanobis + sparse-random
		60–40	70–30	80–20
KAZE _350	HOG + BoE	97.75%	97.97%	98.22%
FAST_ 1500		97.03%	97.66%	98.33%
SURF _2200		96.84%	97.09%	97.24%
BRISK _1200		97.60%	98.08%	98.43%

**Table 4:** Results of bag of expressions-based VMMR approach with the different coding design models.

Feature Extraction		Distance Measure	Average Accuracy % with Coding Design		
			One-vs.-all	All-pairs	Sparse-random
KAZE_350	HOG + BoE	Mahalanobis	98.27%	98.00%	98.22%
		Euclidean	98.19%	97.83%	98.18%
FAST_1500		Mahalanobis	98.22%	97.09%	98.33%
		Euclidean	98.36%	96.85%	98.21%
SURF_2200		Mahalanobis	97.15%	96.32%	97.24%
		Euclidean	97.32%	96.41%	97.19%
BRISK_1200		Mahalanobis	98.49%	97.37%	98.43%
		Euclidean	98.35%	97.66%	98.25%

**Table 5:** The average per-class accuracy of the proposed approaches on the NTOU-MMR dataset. The last column shows the mean average precision of vehicle classes.

**Method**		**C1**	**C2**	**C3**	**C4**	**C5**	**C6**	**C7**	**C8**	**C9**	**C10**	**C11**
KAZE_350	HOG + BoE	99.6	99.4	98.4	99.1	100	97.9	99.3	99.7	100	90.8	90.4
FAST_1500		99.4	100	97.8	100	99.8	100	100	99.5	100	94.6	99.6
SURF_2200		98.1	100	97.9	99.6	100	99.7	99.4	98.5	100	87.5	82.4
BRISK_1200		99.1	100	99.6	100	100	100	98.7	98.3	100	90.8	89.6
**Method**		**C12**	**C13**	**C14**	**C15**	**C16**	**C17**	**C18**	**C19**	**C20**	**C21**	**C22**
KAZE_350	HOG + BoE	100	100	99.7	99.4	98.5	99.3	99.8	100	99.5	99.2	99.7
FAST_1500		100	100	100	99.6	98.4	99.4	99.7	99.5	100	99.7	86.7
SURF_2200		100	96.9	100	100	99.6	99.8	100	100	99.7	100	91.2
BRISK_1200		100	100	100	89.8	98.4	100	100	95.6	100	100	99.6
**Method**		**C23**	**C24**	**C25**	**C26**	**C27**	**C28**	**C29**	**Average per-class Accuracy**			
KAZE_350	HOG + BoE	95.7	100	89.6	99.6	99.4	94.4	100	98.22%			
FAST_1500		99.6	99.7	90.9	99.5	91.9	96.3	100	98.33%			
SURF_2200		100	100	99.3	100	73.9	95.6	100	97.24%			
BRISK_1200		96.1	100	99.7	100	99.7	99.5	100	98.43%			

**Table 6:** Comparison with state-of-the-art work.

Work/Hardware	Features		Classification	Dataset	Average Accuracy	Speed (fps)
Baran et al. [20] (2015) CPU—Dual Core i5 650 CPU 3200 MHz 2GB RAM Win. Server 2008 R2 Enterprise (64-bit).	SURF, SIFT, Edge-Histogram		Multi-class SVM	3859 vehicle images with 17 classes	91.70% 97.20%	30 0.5
He et al. [23] (2015) Hardware unknown	Multi-scale retinex		Artificial Neural Network	1196 vehicle images and 30 classes	92.47%	1
Tang et al. [30] (2017) i7 3.4GHz, 4GB RAM Quadro 2000 GPU	Local Gabor Binary Pattern		Nearest Neighborhood	223 vehicle images with 8 classes	91.60%	3.3
Nazemi et al. [40] (2018) 3.4 GHz Intel CPU 32 GB RAM	Dense-SIFT		A fine-grained classification	Iranian on-road vehicle dataset	97.51%	11.1
Jie Fang et al. [31] (2017) ı7-4790K CPU TITAN X GPU			Convolution Neural Network	44,481 vehicle images with 281 classes	98.29%	–
Afshin Dehghan et al. [32] (2017) TITAN XP GPUs			Convolution Neural Network	44,481 vehicle images with 281 classes	95.88%	–
Hyo Jong et al. [41] (2019) i7-4790 CPU 3.6GHz GTX 1080 GPU			Residual Squeeze Net	291,602 vehicle images with 766 classes	96.33%	9.1
Chen et al. [18] (2015) Hardware unknown	Symmetric SURF		Sparse representation and hamming distance	NTOU-MMR	91.10%	0.46
Manzoor et al. [42] [2017) i7 3.4GHz 16GB RAM	SIFT		SVM	NTOU-MMR	89.00%	–
Jabbar et al. [25] (2016) i5 CPU 2.94GHz 16GB RAM	SURF		Single and ensemble of multi-class SVM	NTOU-MMR	94.84%	7.4
Manzoor et al. [33] [2019) i7 3.4GHz 16GB RAM	HOG		Random Forest SVM	NTOU-MMR	94.53% 97.89%	35.7 13.9
Our Approach i7-4600M CPU 2.90GHz 8GB RAM	KAZE_350	HOG + BoE	Linear SVM	NTOU-MMR	98.22%	24.7
	FAST_1500				98.33%	15.4
	SURF_2200				97.24%	18.6
	BRISK_1200				98.43%	6.7

## References

[1] Pearce G., Pears N. Automatic make and model recognition from frontal images of cars. Proceedings of the 2011 8th IEEE International Conference on Advanced Video and Signal Based Surveillance (AVSS).

[2] Sivaraman S., Trivedi M.M. (2013). Looking at vehicles on the road: A survey of vision-based vehicle detection, tracking, and behavior analysis. IEEE Trans. Intell. Transp. Syst..

[3] Hsieh J.W., Chen L.C., Chen D.Y. (2014). Symmetrical SURF and its applications to vehicle detection and vehicle make and model recognition. Trans. Intell. Transp. Syst..

[4] Tsai L.W., Hsieh J.W., Fan K.C. (2007). Vehicle detection using normalized color and edge map. IEEE Trans. Image Process..

[5] Wang S., Cui L., Liu D., Huck R., Verma P., Sluss J.J., Cheng S. (2012). Vehicle Identification Via Sparse Representation. IEEE Trans. Intell. Transp. Syst..

[6] Kim Z., Malik J. Fast vehicle detection with probabilistic feature grouping and its application to vehicle tracking. Proceedings of the Ninth IEEE International Conference on Computer Vision.

[7] Kumar T.S., Sivanandam S. (2012). A modified approach for detecting car in video using feature extraction techniques. Eur. J. Sci. Res..

[8] Betke M., Haritaoglu E., Davis L.S. (2000). Real-time multiple vehicle detection and tracking from a moving vehicle. Mach. Vis. Appl..

[9] Gu H.Z., Lee S.Y. (2013). Car model recognition by utilizing symmetric property to overcome severe pose variation. Mach. Vis. Appl..

[10] Hoffman C., Dang T., Stiller C. Vehicle detection fusing 2D visual features. Proceedings of the IEEE Intelligent Vehicles Symposium.

[11] Leotta M.J., Mundy J.L. (2010). Vehicle surveillance with a generic, adaptive, 3d vehicle model. IEEE Trans. Pattern Anal. Mach. Intell..

[12] Guo Y., Rao C., Samarasekera S., Kim J., Kumar R., Sawhney H. Matching vehicles under large pose transformations using approximate 3d models and piecewise mrf model. Proceedings of the 2008 IEEE Conference on Computer Vision and Pattern Recognition.

[13] Hou T., Wang S., Qin H. Vehicle matching and recognition under large variations of pose and illumination. Proceedings of the 2009 IEEE Computer Society Conference on Computer Vision and Pattern Recognition Workshops.

[14] Faro A., Giordano D., Spampinato C. (2011). Adaptive background modeling integrated with luminosity sensors and occlusion processing for reliable vehicle detection. IEEE Trans. Intell. Transp. Syst..

[15] Jazayeri A., Cai H., Zheng J.Y., Tuceryan M. (2011). Vehicle detection and tracking in car video based on motion model. IEEE Trans. Intell. Transp. Syst..

[16] Rojas J.C., Crisman J.D. Vehicle detection in color images. Proceedings of the Conference on Intelligent Transportation Systems.

[17] Guo D., Fraichard T., Xie M., Laugier C. Color modeling by spherical influence field in sensing driving environment. Proceedings of the IEEE Intelligent Vehicles Symposium 2000 (Cat. No. 00TH8511).

[18] Chen L.C., Hsieh J.W., Yan Y., Chen D.Y. (2015). Vehicle make and model recognition using sparse representation and symmetrical SURFs. Pattern Recognit..

[19] Lowe D.G. (2004). Distinctive image features from scale-invariant keypoints. Int. J. Comput. Vis..

[20] Baran R., Glowacz A., Matiolanski A. (2015). The efficient real-and non-real-time make and model recognition of cars. Multimedia Tools Appl..

[21] Fraz M., Edirisinghe E.A., Sarfraz M.S. Mid-level-representation based lexicon for vehicle make and model recognition. Proceedings of the 22nd International Conference on Pattern Recognition.

[22] Manzoor M.A., Morgan Y. Vehicle make and model recognition using random forest classification for intelligent transportation systems. Proceedings of the 8th Annual Computing and Communication Workshop and Conference (CCWC).

[23] He H., Shao Z., Tan J. (2015). Recognition of car makes and models from a single traffic-camera image. IEEE Trans. Intell. Transp. Syst..

[24] Bay H., Ess A., Tuytelaars T., Van Gool L. (2008). Speeded-up robust features (SURF). Comput. Vision Image Understanding.

[25] Siddiqui A.J., Mammeri A., Boukerche A. (2016). Real-time vehicle make and model recognition based on a bag of SURF features. IEEE Trans. Intell. Transp. Syst..

[26] Nazir S., Yousaf M.H., Nebel J.C., Velastin S.A. (2019). Dynamic Spatio-Temporal Bag of Expressions (D-STBoE) model for human action recognition. Sensors.

[27] Nazir S., Yousaf M.H., Nebel J.C., Velastin S.A. (2018). A Bag of Expression framework for improved human action recognition. Pattern Recognit. Lett..

[28] Psyllos A., Anagnostopoulos C.N., Kayafas E. (2011). Vehicle model recognition from frontal view image measurements. Comput. Stand. Interfaces.

[29] Varjas V., Tanács A. Car recognition from frontal images in mobile environment. Proceedings of the 8th International Symposium on Image and Signal Processing and Analysis (ISPA).

[30] Tang Y., Zhang C., Gu R., Li P., Yang B. (2017). Vehicle detection and recognition for intelligent traffic surveillance system. Multimed. Tools Appl..

[31] Fang J., Zhou Y., Yu Y., Du S. (2016). Fine-grained vehicle model recognition using a coarse-to-fine convolutional neural network architecture. IEEE Trans. Intell. Transp. Syst..

[32] Dehghan A., Masood S.Z., Shu G., Ortiz E. View Independent Vehicle Make, Model and Color Recognition Using Convolutional Neural Network. https://arxiv.org/abs/1702.01721.

[33] Manzoor M.A., Morgan Y., Bais A. (2019). Real-Time Vehicle Make and Model Recognition System. Mach. Learn. Knowl. Extr..

[34] Soon F.C., Khaw H.Y., Chuah J.H., Kanesan J. (2018). PCANet-based convolutional neural network architecture for a vehicle model recognition system. IEEE Trans. Intell. Transp. Syst..

[35] Alcantarilla P.F., Bartoli A., Davison A.J. (2012). KAZE features. Computer Vision—ECCV 2012.

[36] Rosten E., Drummond T. (2006). Machine learning for high-speed corner detection. Computer Vision—ECCV 2006.

[37] Leutenegger S., Chli M., Siegwart R. BRISK: Binary robust invariant scalable keypoints. Proceedings of the 2011 IEEE international conference on computer vision.

[38] Dalal N., Triggs B. Histograms of oriented gradients for human detection. Proceedings of the 2005 IEEE Computer Society Conference on Computer Vision and Pattern Recognition.

[39] Jain A.K. (2010). Data clustering: 50 years beyond K-means. Pattern Recognit. Lett..

[40] Nazemi A., Shafiee M.J., Azimifar Z., Wong A. Unsupervised Feature Learning Toward a Real-time Vehicle Make and Model Recognition. https://arxiv.org/abs/1806.03028.

[41] Lee H.J., Ullah I., Wan W., Gao Y., Fang Z. (2019). Real-time vehicle make and model recognition with the residual SqueezeNet architecture. Sensors.

[42] Manzoor M.A., Morgan Y. Vehicle Make and Model classification system using bag of SIFT features. Proceedings of the 7th Annual Computing and Communication Workshop and Conference (CCWC).

